# Improving L-arabinose utilization of pentose fermenting *Saccharomyces cerevisiae *cells by heterologous expression of L-arabinose transporting sugar transporters

**DOI:** 10.1186/1754-6834-4-38

**Published:** 2011-10-12

**Authors:** Thorsten Subtil, Eckhard Boles

**Affiliations:** 1 Institute of Molecular Biosciences, Goethe-University Frankfurt am Main, Max-von-Laue-Strasse 9, D-60438 Frankfurt am Main, Germany

## Abstract

**Background:**

Hydrolysates of plant biomass used for the production of lignocellulosic biofuels typically contain sugar mixtures consisting mainly of D-glucose and D-xylose, and minor amounts of L-arabinose. The yeast *Saccharomyces cerevisiae *is the preferred microorganism for the fermentative production of ethanol but is not able to ferment pentose sugars. Although D-xylose and L-arabinose fermenting *S. cerevisiae *strains have been constructed recently, pentose uptake is still a limiting step in mixed sugar fermentations.

**Results:**

Here we described the cloning and characterization of two sugar transporters, AraT from the yeast *Scheffersomyces stipitis *and Stp2 from the plant *Arabidopsis thaliana*, which mediate the uptake of L-arabinose but not of D-glucose into *S. cerevisiae *cells. A yeast strain lacking all of its endogenous hexose transporter genes and expressing a bacterial L-arabinose utilization pathway could no longer take up and grow with L-arabinose as the only carbon source. Expression of the heterologous transporters supported uptake and utilization of L-arabinose especially at low L-arabinose concentrations but did not, or only very weakly, support D-glucose uptake and utilization. In contrast, the *S. cerevisiae *D-galactose transporter, Gal2, mediated uptake of both L-arabinose and D-glucose, especially at high concentrations.

**Conclusions:**

Using a newly developed screening system we have identified two heterologous sugar transporters from a yeast and a plant which can support uptake and utilization of L-arabinose in L-arabinose fermenting *S. cerevisiae *cells, especially at low L-arabinose concentrations.

## Background

Lignocellulosic biomass represents the most important renewable resource that can be used for the production of biofuels, after its biological conversion into ethanol. D-glucose is the most abundant hexose sugar in lignocellulosic biomass. It can be efficiently fermented to ethanol by the yeast *Saccharomyces cerevisiae *with yields close to the theoretical maximum [1]. D-xylose and L-arabinose are the major five-carbon sugars present in biomass hydrolysate streams. Unfortunately, wild-type *S. cerevisiae *is unable to utilize these pentose sugars as fermentative substrates. However, for economically feasible fermentation processes, the bioconversion of all sugars in the raw material is essential.

To overcome this limitation, heterologous pentose utilization pathways from pentose-assimilating organisms have been introduced into *S. cerevisiae*, allowing fermentation of D-xylose and L-arabinose [2-7]. Yet, an efficient uptake of pentose sugars into the yeast cells is still a limiting factor for the co-fermentation of sugar mixtures as found in biomass hydrolysates. Simultaneous uptake and fermentation of hexose and pentose sugars is a prerequisite to allow accelerated overall fermentation.

Interestingly, both pentose sugars, although not metabolized by wild-type yeast strains, can be taken up by *S. cerevisiae*. The hexose transporters of *S. cerevisiae*, especially Hxt7, Hxt5 and Gal2, catalyze uptake of D-xylose [8-10] and Gal2 also mediates the transport of L-arabinose [11]. However, uptake of pentoses by hexose transporters occurs only with low affinity and in competition with D-glucose. D-glucose inhibits pentose uptake, and pentose consumption starts only once D-glucose levels have decreased significantly (TS and EB, manuscript in preparation, [12]).

Substantial research efforts have been made in attempting to identify specific heterologous pentose transporters for functional expression in *S. cerevisiae*. In contrast to many bacterial enzymes, heterologously expressed bacterial transporters do not support the uptake of sugars into yeast cells as most of them are not correctly targeted to the plasma membrane (TS and EB, unpublished results, [8]). Nevertheless, for the uptake of D-xylose, expression of various eukaryotic transporters from *Arabidopsis thaliana*, *Candida intermedia*, *Debaryomyces hansenii*, *Hypocrea jecorina*, *Neurospora crassa *and *Scheffersomyces stipitis *have been reported [9,13-19]. Moreover, for the simultaneous fermentation of D-xylose and cellobiose, a heterologous cellobiose transporter has been expressed together with a cytosolically localized β-glucosidase [20,21].

For L-arabinose uptake, sugar transporters of a few natural L-arabinose metabolizing yeasts like *Candida *spp., *Pichia *spp., *Arxula adeninivorans*, *Debaryomyces hansenii *and *Kluyveromyces marxianus *have been characterized [22-24]. Recently, two genes from *Ambrosiozyma monospora *were reported to encode specific L-arabinose transporters [25]. However, the functional expression of a heterologous L-arabinose transporter in *S. cerevisiae *has not been reported so far. Here, we describe the construction of an L-arabinose transporter screening system based on a *S. cerevisiae *strain without a hexose/pentose transporter expressing an L-arabinose utilization pathway. This strain is able to grow on L-arabinose media only after functional expression of L-arabinose transporters. Using this screening system, we identified and characterized two transporters from *S. stipitis *and *A. thaliana *supporting uptake of and growth with L-arabinose, especially at low L-arabinose concentrations, but not with D-glucose.

## Results

### Construction of an L-arabinose transporter screening system

In the yeast strain EBY.VW4000, there are 17 genes encoding all of the members of the hexose transporter family and three genes encoding maltose/glucose transporters which are deleted [26]. As the strain still contains a specific maltose transporter, it grows normally on maltose medium, but is no longer able to grow with D-glucose, D-fructose or D-mannose and only very slowly with D-galactose as carbon sources [26]. We assumed that the strain is also no longer able to take up pentose sugars like D-xylose and L-arabinose and therefore should be an ideal screening system for heterologously expressed pentose transporters [8]. In this work, we concentrated on the uptake of L-arabinose. As it was shown before that an increased transaldolase activity is crucial for efficient L-arabinose utilization [27], the weak endogenous promoter of *TAL1 *in EBY.VW4000 was exchanged for a strong and constitutive *HXT7 *promoter fragment [8], resulting in strain MKY06. This strain was transformed with plasmids p423H7-synthIso, p424H7-synthKin and p425H7-synthEpi, expressing the enzymes of an optimized bacterial L-arabinose utilization pathway [4], and was named TSY01. In contrast to a wild-type strain, this strain was not able to grow with L-arabinose as the sole carbon source as it cannot take up L-arabinose (see below).

**Figure 1:**
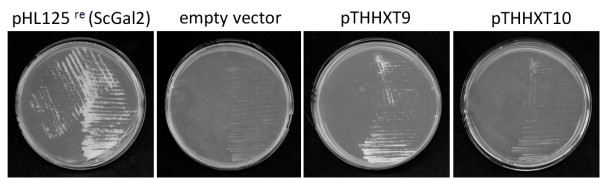
**Growth properties of strain TSY01 overexpressing individual sugar transporters of *S. cerevisiae***. *S. cerevisiae *strain TSY01 lacking all hexose transporters and expressing a bacterial L-arabinose utilization pathway was transformed with plasmids coding for various hexose transporters of *S. cerevisiae *[8]. Transformants were streaked on agar plates with synthetic complete medium with 20 g/L L-arabinose and incubated at 30°C for ten days. Only cells expressing ScGal2, ScHxt9 or ScHxt10 could grow. Cells transformed with the empty vector p426H7-6HIS served as a negative control.

To test which of the yeast hexose transporters were able to support the uptake of L-arabinose, all of them were overexpressed individually in strain TSY01. TSY01 was transformed with a series of multicopy-plasmids overexpressing all of the *S. cerevisiae *hexose transporters from Hxt1 to Hxt17 [8,26], with plasmid pHL125^re ^expressing the yeast Gal2 galactose transporter and with the empty plasmid p426H7-6HIS as a negative control. Transformants were first plated on selective agar plates without uracil, histidine, tryptophan and leucine, and with maltose as a permissive carbon source. The resulting colonies were streaked out on synthetic medium with 20 g/L L-arabinose as the only carbon source. Transformants expressing ScGal2, ScHxt9 and ScHxt10 could grow on the L-arabinose medium after 10 days of incubation at 30°C, with *GAL2*-expressing transformants growing the fastest (Figure 1). All other transformants, including those with the empty vector control, did not grow at all. The results indicate that ScGal2, ScHxt9 and ScHxt10 are the only *S. cerevisiae *transporters enabling uptake of L-arabinose, with ScGal2 being the most effective one. Furthermore, strain TSY01 provides an ideal screening and test system for the investigation and characterization of heterologously expressed L-arabinose transporters.

### Identification of an L-arabinose transporting protein, AraT, from *S. stipitis*

**Figure 2:**
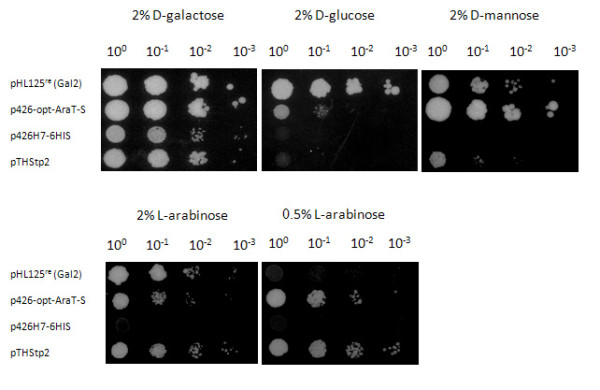
**Growth properties of TSY01 cells expressing different sugar transporters ScGal2 (pHL125^re^), SsAraT (p426-opt-AraT-S) or AtStp2 (pTHStp2)**. Cells were spotted in serial dilutions on synthetic complete medium agar plates with various carbon sources: D-galactose, D-glucose, D-mannose and different L-arabinose concentrations (20 g/L and 5 g/L), and incubated at 30°C for 5 days (D-galactose, D-glucose, D-mannose) or 10 days (L-arabinose). Cells transformed with the empty vector p426H7-6HIS served as a negative control.

The gene library YEpTW had been constructed from partially digested chromosomal DNA of the *S. stipitis *strain CBS5774 [28]. YEpTW was transformed into strain TSY01 and the cells were plated on agar plates with selective medium containing 20 g/L maltose. The colonies obtained after three days of incubation at 30°C were replica-plated on selective medium agar plates with either 20 g/L L-arabinose or 20 g/L D-glucose. After 10 days at 30°C, some colonies exhibited growth on both the D-glucose and the L-arabinose plates, but two colonies did only grow on the L-arabinose plates. Re-isolation of the plasmids and sequencing of the inserted DNA sequences showed that both plasmids carried overlapping fragments of a gene from *S. stipitis *encoding a putative sugar transporter [EMBL:ABN64726]. We called this gene *ARAT*. The coding sequence of SsAraT was codon-optimized according to the glycolytic codon usage of *S. cerevisiae *as described in Wiedemann and Boles [4], and cloned behind the strong and constitutive *HXT7 *promoter fragment on plasmid p426H7-6HIS, resulting in plasmid p426-opt-AraT-S. TSY01 transformants expressing the codon-optimized variant of SsAraT could grow with high (20 g/L) and low (5 g/L) L-arabinose concentrations, with 20 g/L D-galactose or D-mannose, but only slowly with 20 g/L D-glucose as carbon sources (Figure 2). In contrast, ScGal2 supported fast growth with all of the sugars at 20 g/L but no growth with low (5 g/L) L-arabinose concentrations. In a similar screening system with a strain overexpressing the *Clostridium phytofermentans *xylose isomerase [3], SsAraT did not support growth on xylose, in contrast to ScHxt7 and ScGal2, indicating that SsAraT is not able to take up xylose (data not shown).

### Stp2 from *A. thaliana *is a high-affinity D-galactose/L-arabinose transporter

**Figure 3:**
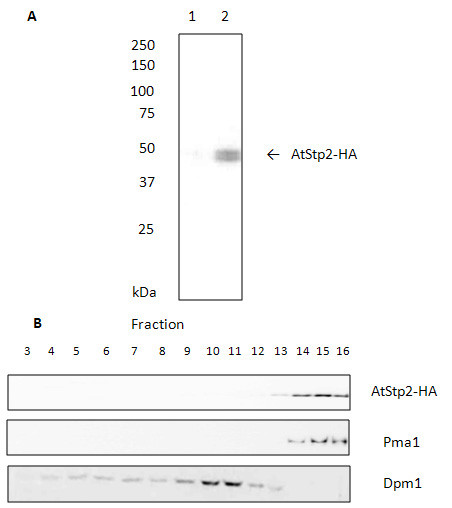
**Western blot analysis and intracellular localisation of AtStp2 in *S. cerevisiae***. **(A) **Crude extracts of L-arabinose-grown TSY01 cells expressing either ScGal2 (lane 1), or HA-tagged AtStp2 (lane 2) were prepared, and 20 μg of total protein were separated by SDS PAGE. Western blotting was performed as described in Methods. The arrow indicates the HA-tagged AtStp2 protein. **(B) **For subcellular localization of AtStp2 crude extract of TSY01 cells grown on L-arabinose expressing HA-tagged AtStp2 was transferred on a seven step sucrose density gradient. After centrifugation the gradient was fractionated and the localization of AtStp2-HA in *S. cerevisiae *was analyzed via Western blot analysis. Antibodies against Pma1 (plasma membrane) and Dpm1 (endoplasmic reticulum) served as controls. HA: hemagglutinin.

The sugar transporter Stp2 from the plant *A. thaliana *was previously characterized as a proton symporter with a high affinity for D-galactose [29]. Due to the fact that the D-galactose transporter Gal2 from *S. cerevisiae *is able to take up L-arabinose as well, AtStp2 should be characterized as a putative L-arabinose transporter in the TSY01 test system. To see whether the AtStp2 protein is produced in *S. cerevisiae *and correctly targeted to the plasma membrane, it was first expressed with a hemagglutinin (HA)-tag at its C-terminus from plasmid p426-Stp2-HA in strain TSY01. AtStp2 could be identified in Western blots as a distinct band with a size of about 50 kDa (Figure 3A). Sucrose density gradient fractionation experiments showed that AtStp2 clearly co-localized with the yeast plasma membrane ATPase Pma1 (Figure 3B), indicating that it is expressed in yeast and localized at the cell surface.

For the growth experiments, plasmid pTHStp2 expressing AtStp2 behind the strong *HXT7 *promoter fragment [8] was transformed into yeast strain TSY01. Transformants were selected on maltose agar plates and tested for growth on various carbon sources in serial dilutions (Figure 2). AtStp2 supported growth of the cells on plates containing high (20 g/L) and low (5 g/L) L-arabinose concentrations, and with D-galactose. In contrast to SsAraT it did not support any growth with D-glucose and only very slow growth with D-mannose (Figure 2). These results indicate that AtStp2 is able to transport L-arabinose but not D-glucose. In the screening system with a strain overexpressing the *C. phytofermentans *xylose isomerase (see above), AtStp2, like SsAraT, did not support growth on D-xylose, indicating that AtStp2 is also not able to take up D-xylose (data not shown).

### Characterization of the influence of SsAraT, AtStp2 and ScGal2 on L-arabinose utilization

**Figure 4:**
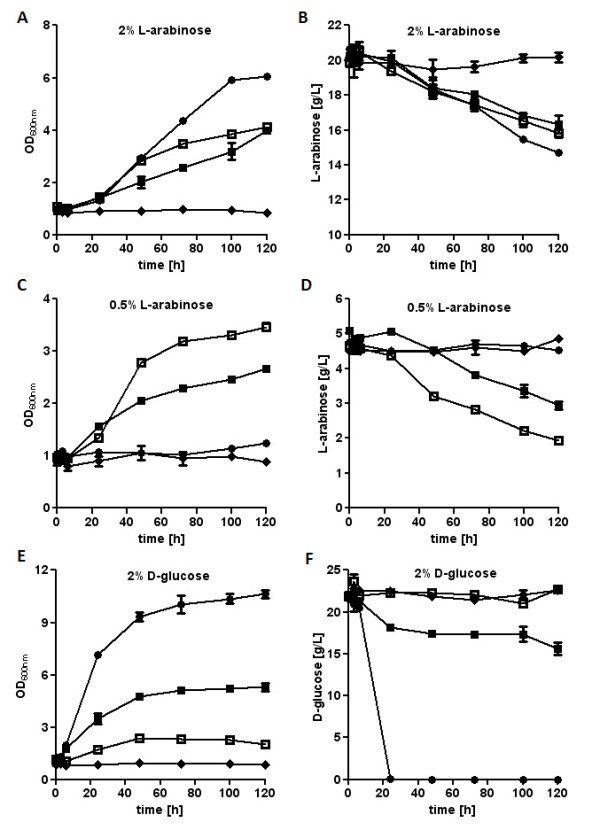
**Growth and sugar utilization in aerobic batch cultures of TSY01 expressing L-arabinose transporters**. Cells expressing ScGal2 (black circle), SsAraT (black square), AtStp2 (open square) or containing the empty vector (black diamond) were incubated in synthetic complete medium with 20 g/L L-arabinose (A/B), 5 g/L L-arabinose (C/D) or 20 g/L D-glucose (E/F) as carbon sources. Growth was determined by measuring the optical density at 600 nm and sugar concentrations were analyzed by high performance liquid chromatography. The results shown are average values for two to three independent cultures.

To demonstrate and compare the efficiencies of SsAraT, AtStp2 and ScGal2 in L-arabinose utilization by recombinant *S. cerevisiae *cells, growth and sugar consumption were characterized in strain TSY01 expressing the various transporters by shake-flask aerobic batch cultivations. Precultures of the strains expressing the L-arabinose transporters were obtained in SC medium with 20 g/L L-arabinose, while the control strain with the empty vector p426H7-6HIS was pregrown in synthetic complete (SC) medium with 10 g/L maltose. Cells were harvested, washed and inoculated in 50 mL SC medium containing high (20 g/L) or low (5 g/L) L-arabinose concentrations or 20 g/L D-glucose. Growth performance and consumption of sugars were investigated under aerobic conditions (Figure 4). TSY01 containing the empty vector did not grow nor did it utilize the sugars under any conditions. With an L-arabinose concentration of 20 g/L, all three L-arabinose transporters supported growth of the cells, with ScGal2 supporting slightly higher growth rates than the other transporters (Figure 4A-B). On 5 g/L L-arabinose, cells expressing ScGal2 could hardly grow at all, whereas those expressing SsAraT and AtStp2 grew well, with those expressing AtStp2 growing the fastest. These findings were confirmed by the analysis of sugar consumption (Figure 4C-D). All strains expressing the L-arabinose transporters consumed L-arabinose at 20 g/L. However, only the strains expressing AtStp2 and SsAraT, but not those expressing ScGal2, consumed L-arabinose at a concentration of 5 g/L.

While ScGal2 enabled the cells to efficiently grow on D-glucose and to consume all of the D-glucose in less than 25 hours, cells expressing SsAraT grew only slowly and consumed only minor amounts of the D-glucose. In contrast, cells expressing AtStp2 did not consume any D-glucose and showed only residual growth on D-glucose, which probably resulted from the consumption of storage carbohydrates (Figure 4E-F). These results demonstrate that SsAraT and AtStp2 support the efficient uptake of L-arabinose but not of D-glucose into yeast cells, and do so especially at low L-arabinose concentrations, in contrast to ScGal2.

### Analyses of sugar uptake mediated by SsAraT, AtStp2 and ScGal2

**Figure 5:**
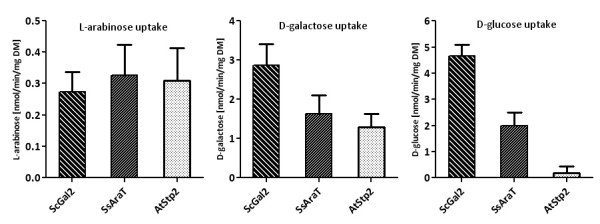
**Initial rates of sugar uptake of strain MKY06 expressing AtStp2, SsAraT or ScGal2**. Cells were grown on D-galactose or maltose (empty vector), harvested and incubated with radioactively labeled sugars (10 mM L-arabinose, 10 mM D-galactose, 10 mM D-glucose) for 2 minutes (L-arabinose) or 1 minute (D-galactose, D-glucose). The background values determined with cells containing the empty vector were subtracted.

To directly determine and compare the specificities of the L-arabinose transporters, the initial rates of sugar uptake in strain MKY06 (without the L-arabinose utilization pathway) overexpressing SsAraT, AtStp2 or ScGal2 were measured with radioactively labeled sugars (Figure 5). Cells were pre-grown in SC medium with D-galactose (cells expressing transporters) or maltose (empty vector). L-arabinose, D-galactose and D-glucose uptake rates were measured during 1- or 2-minute time intervals, respectively, at 10 mM final sugar concentrations. Uptake of D-galactose was mediated by all three transporters. Whereas a high rate of D-glucose uptake could be measured with cells expressing ScGal2 (4.7 nmol/min/mg dry mass (DM)), SsAraT mediated only lower rates of D-glucose uptake (2 nmol/min/mg DM), and D-glucose uptake mediated by AtStp2 was hardly to detect (< 0.01 nmol/min/mg DM).

For L-arabinose, uptake kinetics were determined by measuring L-arabinose uptake at various concentrations between 0.1 and 50 mM during 2-minute time intervals. While ScGal2 turned out to transport L-arabinose with low affinity and high capacity, SsAraT and AtStp2 mediated uptake of L-arabinose with low capacity but high affinity (Table 1). In all three cases, addition of 10 mM D-galactose or D-glucose nearly completely inhibited L-arabinose uptake.

**Table 1:** K_M _and v_max _values for D-galactose/L-arabinose transporters

Transporter	ScGal2	SsAraT	AtStp2
**K_M _(mM)**	57 +/-11	3.8 +/- 1.7	4.5 +/- 2.2
**v_max _(nmol/min/mg DM)**	2.2 +/- 0.26	0.4 +/- 0.06	0.6 +/- 0.08

**K_M_: **Michaelis constant; **v_max _:** maximal enzyme reaction velocity

## Discussion

*S. cerevisiae *is not able to utilize the pentose sugars D-xylose and L-arabinose. Nevertheless, pentose utilization pathways from bacteria and fungi have been expressed in *S. cerevisiae*, enabling the yeast cells to utilize and ferment D-xylose and L-arabinose [3-7,30]. However, yeast cells do not have own pentose transporters and the uptake of the pentoses into the yeast cells is mediated unspecifically and with low efficiencies by some members of the huge family of hexose transporters (ScHxt1-17, ScGal2) [8-11]. In this work we could show for the first time that mainly ScGal2, but also ScHxt9 and ScHxt10, can support uptake of L-arabinose if overexpressed. However, these transporters are hardly expressed under normal fermentation conditions on sugar mixtures containing D-glucose [31]. Therefore, especially in the presence of D-glucose or at low pentose concentrations, uptake becomes limiting for pentose utilization. Bacteria exhibit specific uptake systems for D-xylose and L-arabinose [32-35] but functional expression of bacterial sugar transporters in yeast is difficult as most of them are not correctly incorporated into the membrane or are not targeted to the plasma membrane (TS and EB, unpublished results). Specific eukaryotic pentose transporters are not known or they also do not enable yeast cells to take up pentoses efficiently in the presence of D-glucose or at low pentose concentrations, for various reasons [15,16,25,36].

Here, we describe cloning and functional expression of two sugar transporters that support efficient uptake of low concentrations of L-arabinose in *S. cerevisiae*. SsAraT is derived from the yeast *S. stipitis *and the corresponding gene was found in a gene library screen. AtStp2 is derived from the plant *A. thaliana *and was already characterized as a D-galactose transporter [29]. Expression of both transporters supported the growth on and utilization of L-arabinose and D-galactose in a hexose transporterless yeast strain expressing a bacterial L-arabinose utilization pathway. However, they did not, or hardly, help yeast cells to utilize D-glucose or D-xylose. Determination of the initial rates of sugar uptake showed that, in *S. cerevisiae*, ScGal2 and SsAraT supported uptake of L-arabinose, D-galactose and D-glucose whereas AtStp2 supported only uptake of L-arabinose and D-galactose but not of D-glucose. Surprisingly, AtStp2 had been reported to support uptake of D-glucose when expressed in *Schizosaccharomyces pombe *[29]. Maybe failure of AtStp2 to enable *S. cerevisiae *to take up D-glucose might be explained by a D-glucose-mediated post-transcriptional inhibitory mechanism in this yeast. Moreover, in the case of SsAraT, the relatively high initial D-glucose uptake rate does not reflect the slow growth of the transformants on D-glucose and the incomplete utilization of D-glucose. Also, in this case, this might be explained by a regulatory mechanism that somehow inhibits or inactivates the transporter in the presence of D-glucose after some hours. Additionally, even the initial uptake of L-arabinose by SsAraT and AtStp2 was strongly impaired by D-glucose. At least for AtStp2 this was rather surprising, as it could not use D-glucose as a substrate.

The determination of L-arabinose uptake kinetics revealed that, whereas ScGal2 turned out to have a relatively low affinity but high capacity for L-arabinose, SsAraT and AtStp2 exhibited higher affinities but lower capacities. These characteristics were clearly reflected in the growth properties of the strains expressing the individual transporters on different L-arabinose concentrations. ScGal2 supported growth on L-arabinose only at high concentrations, reflecting its low affinity; SsAraT and AtStp2 did so especially at low concentrations due to their higher affinities.

Until now, ScGal2 was the only transporter used to increase L-arabinose uptake in recombinant *S. cerevisiae *fermenting L-arabinose. Either targeted overexpression of ScGal2 improved L-arabinose utilization [27] or expression of *GAL2 *was increased by evolutionary engineering of a yeast strain for improved fermentation of L-arabinose [37]. Also in this work, we could show that at high L-arabinose concentrations ScGal2 efficiently catalyzes L-arabinose uptake. Nevertheless, in many sources of plant biomass L-arabinose is present in only minor amounts. Interestingly, the newly discovered L-arabinose transporters supported efficient uptake of L-arabinose especially at low L-arabinose concentrations, in contrast to ScGal2. Unfortunately, as both transporters are inhibited by D-glucose, they are not expected to improve co-fermentation of D-glucose/L-arabinose mixtures. However, they might improve the fermentation of the low L-arabinose concentrations in typical lignocellulosic hydrolysates after the D-glucose has been consumed.

## Conclusions

We have found and characterized two new high-affinity transporters for improved L-arabinose uptake into *S. cerevisiae *cells. Together with the known ScGal2 low-affinity L-arabinose uptake system, this set of transporters should support uptake of L-arabinose at high and low concentrations and should improve fermentations of lignocellulosic hydrolysates by recombinant L-arabinose fermenting *S. cerevisiae *strains.

## Methods

### Strains and media

Yeast strains and plasmids used in this work are listed in Table 2.

**Table 2:** *S. cerevisiae *strains and plasmids used in this study

*S. cerevisiae *strain or plasmid	Relevant genotype	Source or reference
**Strains**		
EBY.VW4000	*MATa leu2-3,112ura3-52 trp1-289 his3-Δ1 MAL2-8c SUC2 Δhxt1-17Δgal2 Δstl1 Δagt1 Δmph2 Δmph3*	[26]
MKY06	*MATa leu2-3,112ura3-52 trp1-289 his3-Δ1 MAL2-8c SUC2 Δhxt1-17Δgal2 Δstl1 Δagt1 Δmph2 Δmph3 promTAL1::loxP-prom-vkHXT7*	This work
**Plasmids**		
pUG6-kpHXT7	DNA-template for amplification of *kanMX *gene with shortened *HXT7 *promoter for promoter substitution	[41]
pSH47	Cre-recombinase under control of *GAL1 *promoter, *URA3 *marker gene	[40]
pTHStp2	2μ plasmid expressed with the *A. thaliana STP2 *under control of shortened *HXT7 *promoter, *URA3 *marker gene	[8]
p423H7-synthIso	Codon-optimized *Bacillus licheniformis *araA in p423H7-6HIS	[4]
p424H7-synthKin	Codon-optimized *E. coli *araB in p424H7-6HIS, mutation in araB	[4]
p425H7-synthEpi	Codon-optimized *E. coli *araD in p425H7-6HIS	[4]
p426H7-6HIS	2μ plasmid, *URA3 *marker gene	[8]
pHL125^re^	2μ plasmid with the *GAL2 *gene expressed under control of *ADH1 *promoter, *URA3 *marker gene, re-isolated	[47,27]
p426-opt-AraT-S	2μ plasmid with the codon-optimized *S. stipitis ARAT *under control of shortened *HXT7 *promoter, *URA3 *marker gene	This work
p426-Stp2-HA	2μ plasmid expressed with a c-terminal, HA-tagged, full length version of the *A. thaliana STP2 *under control of shortened *HXT7 *promoter, *URA3 *marker gene	This work
pTHHXT9	2μ plasmid with the *HXT9 *gene expressed under control of shortened *HXT7 *promoter, *URA3 *marker gene	[8]
pTHHXT10	2μ plasmid with the *HXT10 *gene expressed under control of shortened *HXT7 *promoter, *URA3 *marker gene	[8]

HA; hemagglutinin.

In aerobic batch cultivations, *S. cerevisiae *was grown in SC medium (1.7 g/L Difco yeast nitrogen base without amino acids and 5 g/L ammoniumsulfate), supplemented with amino acids but omitting the selective plasmid marker nutrients as described previously [38], containing various carbon sources.

For serial dilution growth assays, cells growing in the exponential phase were collected and resuspended in sterile water to an optical density at 600 nm of 1. Cells were serially diluted in 10-fold steps, and 5 μL of each dilution was spotted on agar plates. In aerobic batch cultivations, *S. cerevisiae *was grown in SC medium supplemented with maltose, D-glucose or L-arabinose as carbon sources and buffered at pH 6.3 with 20 mM potassium dihydrogen phosphate. Plasmids were amplified in *Escherichia coli *strain DH5α (Gibco BRL, Gaithersburg, MD) or strain SURE (Stratagene, La Jolla, CA). *E. coli *transformations were performed via electroporation according to the methods of Dower *et al. *[39]. *E. coli *was grown on Luria-Bertani medium with 40 μg/mL ampicillin for plasmid selection.

### Construction of MKY06

The exchange of the endogenous promoter of *TAL1 *in EBY.VW4000 for the shortened *HXT7 *promoter was carried out with a modified *loxP::kanMX::loxP*/Cre recombinase system [40]. A *loxP::kanMX::loxP*-*kpHXT7 *replacement cassette from the plasmid pUG6-kpHxt7 [41] was amplified by PCR using primers S1-pTAL1 (5'-GATGGTGACAAGTGTATAAGTCCTCATCGGGACAGCTACGATTTCTCTTCGTACGCTGCAGGTC

GACGGGAAGAGAGA-3') and S2-pTAL2 (5'-CTAGAGAGTTGTTAGCAACCTTTTGTTTCTTTTGAGCTGGTTCAGACATTTTTTGATTAAAATTA

AAAAAAC-3') (obtained from Eurofins MWG Operon, Ebersberg, Germany).

Yeast transformations were carried out as described previously [42]. As induction of the D-galactose-inducible, D-glucose-repressible Cre recombinase on plasmid pSH47 by D-galactose appeared to have deleterious effects on cells containing several *loxP *sites, we routinely used maltose (which has a weaker repressive effect than D-glucose) to induce/derepress *loxP*-Cre recombination.

### Plasmid construction

A synthetic codon-optimized gene version of AraT from *S. stipitis *was obtained from Sloning BioTechnology (Puchheim, Germany) by changing the original codons to those used in the highly expressed genes encoding glycolytic enzymes in *S. cerevisiae *[4]. Because of a different codon usage of *S. stipitis*, the codon of Serin_407 _was adapted for the usage of *S. cerevisiae*. The coding region of SsAraT with the optimized codon sequence was amplified and cloned into the vector p426H7-6HIS by recombination cloning [26] omitting the six histidine codons. Furthermore, the coding region of Stp2 from *A. thaliana *was amplified from pTHStp2 by PCR and cloned by recombination cloning into the vector p426H7-6HIS, fusing a HA-epitope (YPYDVPDYA) at the C-terminal end of AtStp2 but omitting the six histidine codons. Molecular techniques were performed according to published procedures [43].

### Growth assays

Cultures (50 mL) were grown in 300-mL shake flasks (Erlenmeyer flasks) at 30°C in a shaker. Precultures were grown in SC medium containing 20 g/L L-arabinose or 10 g/L maltose. Cells were washed with sterile water and inoculated to an optical density at 600 nm of 1. All growth assays were carried out at least in duplicate or triplicate.

### Sugar analyses

The concentrations of D-glucose and L-arabinose were determined by HPLC (Dionex BioLC) using a Nugleogel Sugar 810 H exchange column (Macherey-Nagel GmbH & Co, Düren, Germany). The column was eluted at the temperature of 65°C with 5 mM sulfuric acid as a mobile phase with a flow rate of 0.6 mL/min. Detection was done by means of a Shodex RI-101 refractive-index detector (Showa Denko Europe GmbH, Munich, Germany). Chromeleon software 6.50 (Dionex, Idstein, Germany) was used for data evaluation.

### Subcellular localization and Western blot analyses

Yeast transformants expressing a C-terminally HA epitope-tagged variant of AtStp2 and, as a control, those expressing ScGal2 were cultivated until early exponential growth phase in SC medium with L-arabinose, harvested and disrupted with glass beads (0.45 mm) using a Vibrax cell disrupter (Vibrax VXR; Janke & Kunkel (IKA^®^), Staufen, Germany). The protein content was determined according to the method of Bradford [44] and adjusted for equal loading on SDS-PAGE. Twenty micrograms of total protein were loaded in each lane. For Western blot analysis, proteins were transferred from the SDS-PAGE gels to PVDF membranes by submerse electroblotting. AtStp2-HA proteins were detected with rat anti-HA antibody (Roche Diagnostics GmbH, Mannheim, Germany) and goat anti-rat immunoglobulin G coupled to peroxidase (DIANOVA GmbH, Hamburg, Germany). For subcellular localization, the crude extract was loaded on top of a sucrose density gradient [45]. The gradient was generated using the following steps: 1.5 mL 60%, 1.0 mL 37%, 1.5 mL 34%, 2.0 mL 32%, 2.0 mL 29%, 1.5 mL 27%, and 1.0 mL 22%. Pma1 and Dpm1 proteins were detected with mouse anti-Pma1 antibody (Santa Cruz Biotechnology, Inc., Heidelberg, Germany), mouse anti-Dpm1 antibody (Santa Cruz Biotechnology) and rabbit anti-mouse immunoglobulin G coupled to peroxidase (Roche Diagonstics GmbH).

### Sugar uptake analyses

The initial rates of sugar uptake were measured using a modification of the method described by Bisson and Fraenkel [46]. A 50-μL aliquot of a sugar solution containing (1-^3^H)-labeled L-arabinose, (U-^14^C) labeled D-glucose (American Radiolabeled Chemicals Inc., St. Louis (MO), USA) or (1-^14^C) labeled D-galactose (Radiochemical Centre, Amersham, England) was incubated at 30°C and was mixed with 100 μL of yeast suspensions having the same temperature, resulting in final sugar concentrations of 10 mM L-arabinose, D-glucose and D-galactose. For determination of L-arabinose uptake kinetics 0.1, 1, 5, 10 and 50 mM L-arabinose were used. After different time intervals, 10 mL of ice-cold 100 mM potassium phosphate buffer at pH 6.5 with 500 mM D-glucose was added, and the suspension was immediately filtrated using Durapore^® ^membrane filters 0.22 μm pore size (Millipore, Billerica (MA), USA). The filter was washed two times with 10 mL of cold potassium phosphate buffer with 500 mM D-glucose. Filters were transferred to 5 mL scintillation vials containing 4.5 mL Rotiszint^® ^eco plus (Roth, Karlsruhe, Germany) and the radioactivity measured in a scintillation counter. Uptake of radioactivity was nearly linear in time intervals up to 2 minutes. The results shown are average values for two to three independent experiments. Dry weight was determined by filtering 10 mL of the culture through a pre-weighted nitrocellulose filter (0.45 μm pore size; Roth). The filters were washed with demineralized water, dried in a microwave oven for 20 minutes at 140 W, and weighted again. K_M _(Michaelis constant) and v_max _(maximal enzyme reaction velocity) values were calculated using the program GraphPad Prism 5.0 (GraphPad Software, Inc., La Jolla, USA).

## Abbreviations

DM: dry mass; HA: hemagglutinin; HPLC: high perfomance liquid chromatography; kDA: kiloDaltons; PCR: polymerase chain reaction; SC: synthetic complete; K_M _: Michaelis constant; v_max_: maximal enzyme reaction velocity

## Competing interests

The authors declare competing financial interests.

Goethe-University Frankfurt has filed two patent applications concerning the use of the *S. stipitis *transporter and the use of the *A. thaliana *transporter. EB is named as an inventor on both applications. TS is named as an inventor on the application concerning the use of the *A. thaliana *transporter.

## Authors' contributions

TS designed and performed the experiments and wrote the first draft of the manuscript. EB initiated this work, contributed to experimental design and edited the final manuscript. Both authors read and approved the final manuscript.
